# Predicting the incidence of infectious diarrhea with symptom surveillance data using a stacking-based ensembled model

**DOI:** 10.1186/s12879-024-09138-x

**Published:** 2024-02-26

**Authors:** Pengyu Wang, Wangjian Zhang, Hui Wang, Congxing Shi, Zhiqiang Li, Dahu Wang, Lei Luo, Zhicheng Du, Yuantao Hao

**Affiliations:** 1 Department of Medical Statistics, School of Public Health & Center for Health Information Research & Sun Yat-sen Global Health Institute, Sun Yat-sen University, Guangzhou, China; 2 Department of Infectious Disease Control and Prevention, Guangzhou Center for Disease Control and Prevention, Guangzhou, China; 3 Guangzhou Joint Research Center for Disease Surveillance and Risk Assessment, Sun Yat-sen University & Guangzhou Center for Disease Control and Prevention, Guangzhou, China; 4 Peking University Center for Public Health and Epidemic Preparedness & Response, Beijing, China; 5 Department of Epidemiology & Biostatistics, School of Public Health, Peking University, Beijing, China; 6 Key Laboratory of Epidemiology of Major Diseases (Peking University), Ministry of Education, Beijing, China

**Keywords:** Infectious diarrhea, Prediction model, Ensemble learning, Stacking, Symptom surveillance

## Abstract

**Background:**

Infectious diarrhea remains a major public health problem worldwide. This study used stacking ensemble to developed a predictive model for the incidence of infectious diarrhea, aiming to achieve better prediction performance.

**Methods:**

Based on the surveillance data of infectious diarrhea cases, relevant symptoms and meteorological factors of Guangzhou from 2016 to 2021, we developed four base prediction models using artificial neural networks (ANN), Long Short-Term Memory networks (LSTM), support vector regression (SVR) and extreme gradient boosting regression trees (XGBoost), which were then ensembled using stacking to obtain the final prediction model. All the models were evaluated with three metrics: mean absolute percentage error (MAPE), root mean square error (RMSE), and mean absolute error (MAE).

**Results:**

Base models that incorporated symptom surveillance data and weekly number of infectious diarrhea cases were able to achieve lower RMSEs, MAEs, and MAPEs than models that added meteorological data and weekly number of infectious diarrhea cases. The LSTM had the best prediction performance among the four base models, and its RMSE, MAE, and MAPE were: 84.85, 57.50 and 15.92%, respectively. The stacking ensembled model outperformed the four base models, whose RMSE, MAE, and MAPE were 75.82, 55.93, and 15.70%, respectively.

**Conclusions:**

The incorporation of symptom surveillance data could improve the predictive accuracy of infectious diarrhea prediction models, and symptom surveillance data was more effective than meteorological data in enhancing model performance. Using stacking to combine multiple prediction models were able to alleviate the difficulty in selecting the optimal model, and could obtain a model with better performance than base models.

**Supplementary Information:**

The online version contains supplementary material available at 10.1186/s12879-024-09138-x.

## Background

Infectious diarrhea remains a major public health problem worldwide and is a leading cause of death in children. The GBD 2019 study showed that the proportion of DALYs caused by infectious diarrhea in children under 9 years old ranked the third, following lower respiratory tract infections among infectious diseases [1]. The trend of infectious diarrhea incidence in Mainland China is increasing and especially in 0–4 age group, and the increasing trend might continue due to the changes in pathogen spectrum [2].

Prediction can support the prevention and control of infectious diarrhea. Based on the surveillance data, prediction models can be developed to help implement better measures to reduce the burden of disease. Previous studies have used meteorological data to predict the incidence of infectious diarrhea and were able to provide relatively accurate predictions [3, 4]. In the era after the COVID-19 pandemic, there is an increasing emphasis on predicting infectious diseases. In recent years, in addition to conventional infectious disease surveillance, symptom surveillance has been increasingly used internationally as a complementary method of disease surveillance [5–8]. Using multi-source data to build an intelligent early warning system for infectious diseases is one of the main requirements for China’s current infectious disease monitoring and early warning work [9]. Therefore, it is beneficial to include symptom surveillance data as a part of multi-source data. However, there is still a need for further analysis of symptom surveillance data. For example, few studies have used symptom surveillance data to predict infectious diarrhea incidence, and it is not clear whether symptom surveillance data can improve the prediction of infectious diarrhea incidence.

Despite the widespread use of the autoregressive integrated moving average (ARIMA) model for predicting temporal trends in infectious diarrhea and other infectious diseases [10–13], the main limitation lies in its inability to accurately fit and predict the nonlinear trends. This shortcoming can be attributed to the fact that the model is constructed based on the principle of linear correlation, which is inadequate for capturing complex nonlinear patterns in the data [14]. With the advancements in machine learning, various models such as artificial neural networks, support vector machines (including support vector regression), and decision tree-based methods are being increasingly employed to predict infectious diarrhea together with other infectious diseases. For example, Wang et al. employed artificial neural networks, support vector regression, and random forest regression to predict the weekly incidence of infectious diarrhea in Shanghai, China [3]. Abubakar et al. developed an artificial neural networks-based diarrhea incidence prediction model using a vast dataset consisting of demographic, socioeconomic, and environmental variables [15]. These machine learning models, which effectively extracted information concerning nonlinear trends, provided a further improvement compared to the deficiencies of ARIMA. However, selecting the most suitable model from these machine learning models can be a challenging task, as the optimal model may vary depending on the data and settings used. Actually, different machine learning models may have different advantages in data learning, so using an ensemble learning approach to combine different models can play a complementary role to each other, and also alleviate the challenge of determining an optimal model [16].

The ensemble learning is a technique that combines multiple models to accomplish a specific task. By aggregating multiple models, a better-performing model can be obtained. Building an ensemble model involves two main processes: the selection of a methodology for training the participating base models and choosing a suitable process for combining the models’ outputs. According to different methods of the above two processes, various ensemble learning methods have been developed, including AdaBoost, Bagging, Random Forest, Stacking, and others [17]. Stacking can integrate heterogeneous models together, while AdaBoost, Bagging, and random forest can only integrate homogeneous base models [17]. In addition, stacking does not require a particularly large sample size so that it is more suitable for infectious disease surveillance data [18]. Stacking was successfully used in many fields, such as the prediction of crash injury severity [19], the prediction of prices in the agribusiness area [20], the prediction of influenza incidence [21], the subcellular localization prediction for long non-coding RNAs [22] and others. In general, however, the application of stacking in the field of infectious disease prediction is still relatively rare. While there have been a few studies that applied stacking to influenza prediction [16, 23], more research is required to validate its effectiveness in predicting other infectious diseases such as infectious diarrhea.

The stacking model currently has few applications in the field of infectious disease prediction. The conclusions of this study could provide valuable references. Moreover, in the construction of infectious disease prediction models, the optimal model has different conclusions in different studies. This difficulty could be alleviated by integrating different base models by stacking, and at the same time, a better model could be obtained. In this study, symptom surveillance data in previous weeks were included in the construction of the prediction model, and the predicted values obtained through the model would precede the official statistics. In combination with the advantages of stacking models, we could provide predictive information about future epidemics in advance that would be useful in public health decision-making.

## Methods

### Data sources

Infectious diarrhea is an intestinal infectious disease with diarrhea or vomiting as the main symptom. In China, an infectious diarrhea case, which is clinically diagnosed or etiologically confirmed by any hospital or healthcare institution, is required by law to be reported immediately to the notifiable infectious diseases network direct reporting system. In this study, the weekly number of infectious diarrhea cases in Guangzhou during 2016–2021 was accessed from the Guangzhou center for disease control and prevention (Guangzhou CDC), including all the confirmed infectious diarrhea cases in Guangzhou.

The symptom surveillance system for diarrhea-related infectious diseases in China is based on the number of cases with diarrheal syndromes monitored through gastroenterology outpatient clinics. Specifically, healthcare institutions established gastroenterology outpatient clinics for the patients with symptoms such as diarrhea, vomiting, and other suspected diarrhea-related infectious diseases. The number of visits to these gastroenterology outpatient clinics could reflect the number of cases with diarrheal syndromes. In this study, the weekly number of gastroenterology outpatient visits and the rate of weekly gastroenterology outpatient visits to total outpatient visits (weekly gastroenterology outpatient clinic visit rate) in Guangzhou from 2016 to 2021 were obtained from the Guangzhou CDC, which covered all healthcare institutions with gastroenterology outpatient clinics in Guangzhou.

Daily surface meteorological data of Guangzhou City site including air temperature, minimum air temperature, maximum air temperature, relative humidity, precipitation, and atmospheric pressure from 2016 to 2021 were obtained from the National Centers for Environmental Information. Based on the daily meteorological data, weekly mean air temperature, weekly mean minimum air temperature, weekly mean maximum air temperature, weekly mean atmospheric pressure, weekly mean relative humidity, and weekly mean precipitation were calculated to match the infectious diarrhea data and gastroenterology outpatient clinic data.

The mean number of infectious diarrhea cases from June 2021 to Dec 2021 was only 6 per week, whereas the average number of infectious diarrhea cases per week from Jan 2016 to Dec 2021 was 311 (see Additional file 1: Figure S1 in Supplementary material). From the statistical characteristics of the data, the data of infectious diarrhea cases after June 2021 (weeks 26 to 52 of 2021) could be regarded as outliers. It was presumed that the multiple local outbreaks of COVID-19 in Guangzhou in June 2021 led to these outliers. These may not necessarily reflect the true level of incidence, so we excluded these potentially inaccurate data in modelling, only incorporating the 286 time points from week 1 of 2016 to week 25 of 2021 in modelling. Then the dataset was divided into a training set and a testing set in the ratio of 8:2, with the data from week 1 of 2016 to week 20 of 2020 as the training set and the data from week 21 of 2020 to week 25 of 2021 as the testing set. The training set was used to develop models while the testing set was used for the evaluation of model performance. The training set was further divided into a validation set of 20% in model training, which was mainly used to determine the hyperparameters of the models. When better hyperparameters were obtained, the whole training set data was taken to train the final model.

### Model overview

This study implemented an ensemble method of stacking to build the prediction model for infectious diarrhea. The basic framework of stacking is to develop different base models first, and then integrate the output of all base models through a trained meta-model. The base models in this study included artificial neural networks (ANN), Long Short-Term Memory networks (LSTM), support vector regression (SVR) and extreme gradient boosting regression trees (XGBoost). These models are commonly used machine learning models, and they represent several different categories of machine learning models: neural networks, support vector kernel methods, and decision tree-based methods. Another extreme gradient boosting model with linear boosters was used as the meta-model. Different base models have different characteristics, so that we can therefore benefit from the diversity of base models when using stacking method, which is one of the theoretical bases why stacking ensemble learning can improve the prediction performance [17, 24, 25]. However, there is no particular criterion for the number of base models and for choosing base models, as long as the chosen base models are able to achieve the target task. Therefore, considering that choosing too many base models would increase the training time, the above four base models were chosen.

This study developed the predictive model of weekly number of infectious diarrhea cases based on the theory of time series prediction. It used the historical observations of weekly number of infectious diarrhea cases, outpatient variables (the weekly number of gastroenterology outpatient visits and weekly gastroenterology outpatient clinic visit rate) and weekly meteorological variables as inputs to predict the future values of weekly number of infectious diarrhea cases. The task of time series prediction is as follows [26]:

1 $$\eqalign{{{\hat Y}_{{\rm{t + d}}}} =  & H(yt - n + 1, \ldots ,yt - 1,yt,x_{t - n + 1}^1, \ldots , \cr & x_{t - 1}^1,x_t^1,x_{t - n + 1}^2, \ldots ,x_{t - 1}^2,x_t^2, \ldots ,x_{t - n + 1}^L, \ldots ,x_{t - 1}^L,x_t^L) \cr} $$

where $$ H(\bullet )$$ denotes a learner. $$ {y}_{t-n+1},\dots,{y}_{t-1},{y}_{t}$$ denote the historical data points of weekly number of infectious diarrhea cases. *n* denotes the number of historical data points, which is also the time lags. $$ {\widehat{Y}}_{t+d}$$ is the predicted value, which means a *d*-step-ahead prediction. In this research, we set $$ d=1$$ which indicated a one-step-ahead prediction (i.e., one-week-ahead prediction). *y* denoted the weekly number of infectious diarrhea cases, and $${x^1},{x^2}, \ldots,{x^L}$$ referred to other predictors: symptom surveillance data and meteorological variables. *n* and *L* were determined through feature selection. *L* denotes the number of related variables. $$x_t^1,x_t^2, \ldots,x_t^L$$ denote the values of other predictors at timestamp *t*.

### Model evaluation method and metrics

This study evaluated the predictive performance of the models by comparing the evaluation metrics MAPE, RMSE and MAE, which were computed using the testing set data. The evaluation metrics used in this study include: root mean square error (RMSE), mean absolute error (MAE) and mean absolute percentage error (MAPE). Models with better prediction performance have lower RMSE, MAE, and MAPE. MAPE would be used as the most important indicator because the mean value of the weekly number of infectious diarrhea cases was not stable and relative errors could better reflect the predictive performance. The formulas are as follows:

2 $$MAPE=\frac{1}{n}\mathop \sum \nolimits_{{i=1}}^{n} \left| {\frac{{{y_t} - {{\hat {y}}_t}}}{{{y_t}}}} \right| \times 100\% $$

3 $$RMSE = \sqrt {{1 \over n}\sum\nolimits_{i = 1}^n {({y_t}}  - {{\hat y}_t}{)^2}} $$

4 $$MAE=\frac{1}{n}\mathop \sum \nolimits_{{i=1}}^{n} \left| {{y_t} - {{\hat {y}}_t}} \right|$$

where $$ {y}_{t}$$ denotes the observed value, and $$ {\widehat{y}}_{t}$$ is the predicted value. All the calculations of these metrics were based on the testing set.

### Feature selection and model training details

The features included the weekly number of infectious diarrhea cases, gastroenterology outpatient visits, gastroenterology outpatient clinic visit rate, and the meteorological variables in previous *n* weeks according to the results of the autocorrelation and cross-correlation analyses. We considered the temporal characteristics of each variable, the auto-correlation coefficients, and the cross-correlation functions to initially determine the range of *n* (the time lags) and *L* (the number of related variables). We then built the models using different *n* values and incorporating different variables. The feature selection was determined by comparison of the model evaluation metrics.

Based on the stacking framework, we first constructed four base models: ANN, LSTM, SVR, and XGBoost. In the training of ANN, LSTM, and SVR, the datasets were normalized, while XGBoost used the original data directly for training [27]. For the ANN model, the number of neurons in the input layer was based on the value of n and L described above. The number of neurons in the output layer was 1. We set 3 main different structures: (1) 3 dense layers with 64 neurons each; (2) 4 dense layers with 64 neurons each; (3) 5 dense layers with 64 neurons each. For the LSTM model, the number of neurons in the input layer was similarly determined by *n* and *L*. There was 1 neuron in the output layer. Three main different structures were set: (1) 1 LSTM layer with 64 LSTM neurons, and 2 dense layers with 64 neurons each; (2) 1 LSTM layer with 64 LSTM neurons, and 3 dense layers with 64 neurons each; (3) 1 LSTM layer with 64 LSTM neurons, and 4 dense layers with 64 neurons each. Both the structures of the ANN and LSTM models were eventually determined according to the model evaluation metrics calculated.

For the SVR model, a Gaussian kernel was used as the kernel function. The grid search method was used to search for better values of the regularization constant C, and the tolerance error ε parameter. For the XGBoost model, the hyperparameters *n_estimators* and *max_depth* were determined through the validation set divided from the training set. The final XGBoost model was obtained by the whole training set.

**Fig. 1:**
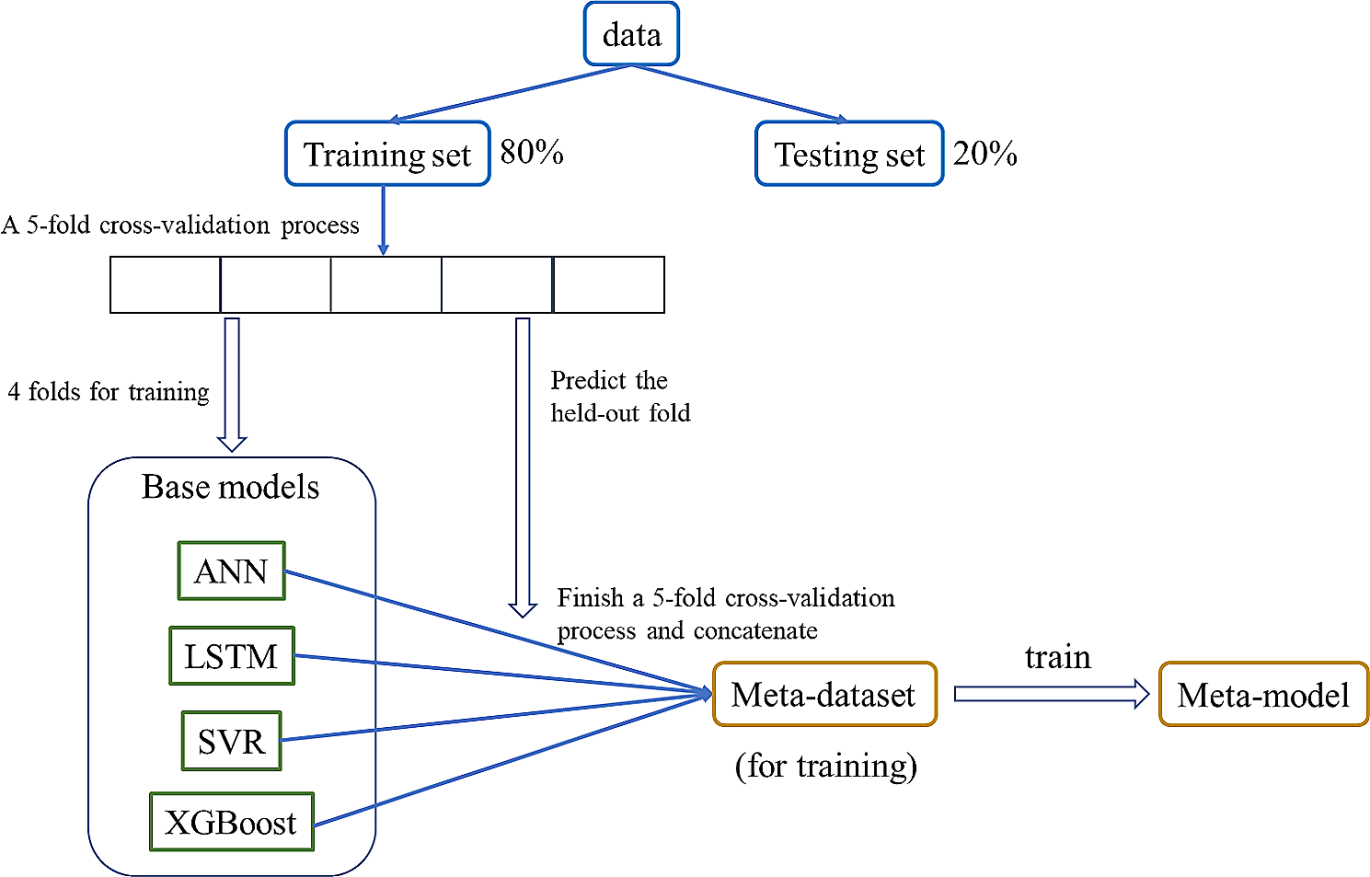
The meta-model training in stacking framework

After building the four base models, we used the base models to generate the meta-data for training the meta-model. It was the most critical step of stacking ensemble. It could also be regarded as the extraction and transformation of the original data features. To avoid overfitting, we further used a 5-fold cross-validation process to generate the subset for training in the meta-data, which was used to develop the meta-model (Fig. 1). Firstly, the training set from the original data was separated into 5 folds. Then we held out one of the folds and trained multiple independent base models using the other folds, and predicted the held-out fold using the base models trained here. After finishing 5 times of the above progress, we could obtain out-of-sample predictions of all 5 folds, which had the same length as the original data. These data formed the inputs of the training set in the meta-data, which was used to train the meta-model. Finally, both the base models and the stacking model were then evaluated using the testing set.

All the analyses were conducted in Python (version 3.9.12). The training of ANN and LSTM was based on Keras (version 2.11.0) and Tensorflow (version 2.11.0). SVR and XGBoost were trained using scikit-learn (version 1.2.2) and xgboost (version 1.7.3), respectively.

## Results

### Descriptive statistics

The average weekly number of infectious diarrhea cases in Guangzhou from January 2016 to December 2021 was 311, with peaks at the end and beginning of each year and low levels of cases during the rest time of the year (see Additional file 1: Figure S1 in Supplementary material). There were elevated outbreak peaks in early 2017 and early 2018. From week 26 to week 52 in 2021, the weekly number of cases was significantly lower than the previous average, which was only 6 cases per week.

The average weekly number of gastroenterology outpatient clinic visits was 1270, and the average weekly gastroenterology outpatient clinic visit rate was 0.48% in Guangzhou from 2016 to 2021. It indicated that the peaks and trends of weekly number of gastroenterology outpatient clinic visits and weekly gastroenterology outpatient clinic visit rate were similar with those of weekly number of infectious diarrhea cases (see Additional file 1: Figure S1 in Supplementary material).

**Fig. 2:**
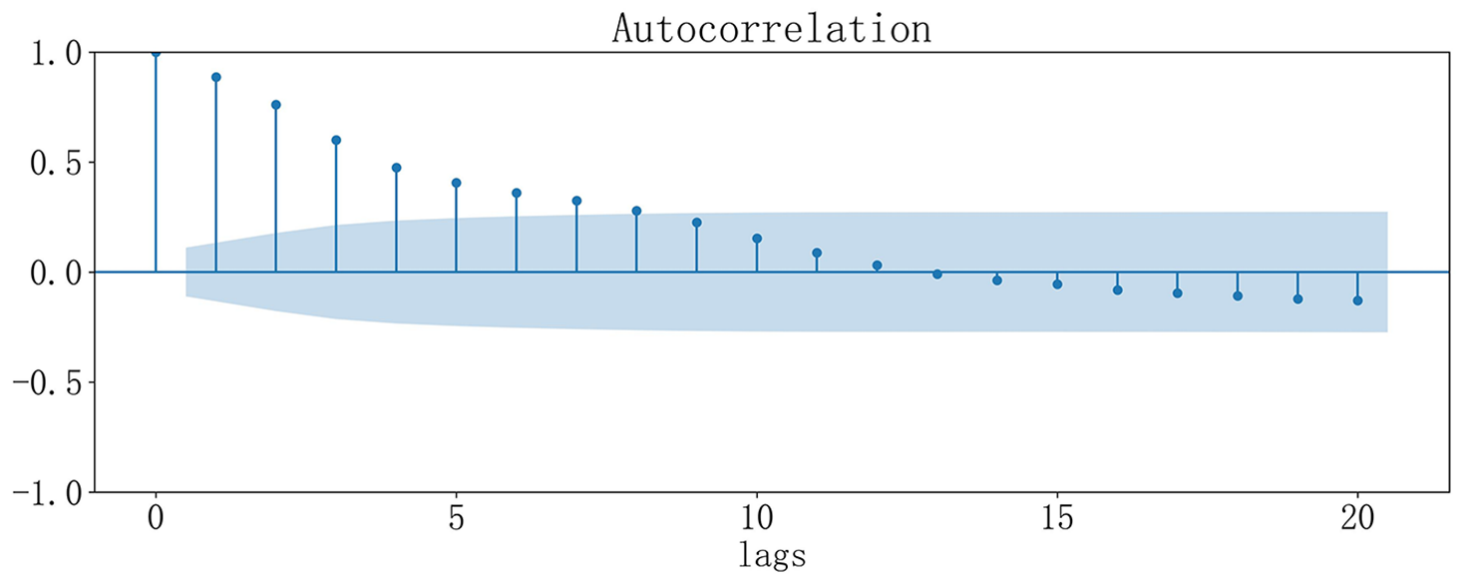
Autocorrelation plot of weekly number of infectious diarrhea cases

**Fig. 3:**
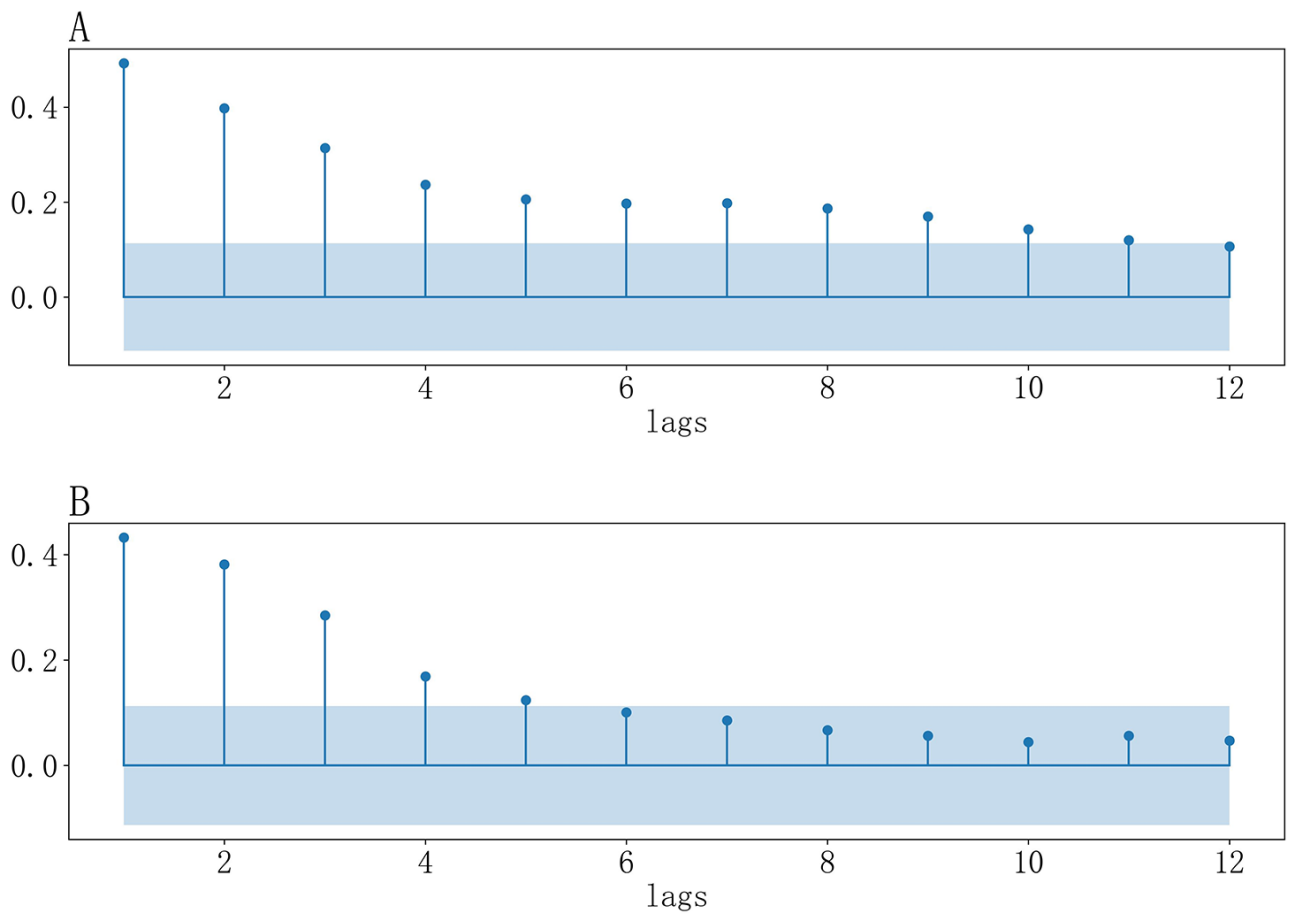
(A-B): (**A**) Cross-correlation coefficients between weekly number of infectious diarrhea cases and weekly number of gastroenterology outpatient clinic visits (**B**) Cross-correlation coefficients between weekly number of infectious diarrhea cases and weekly gastroenterology outpatient clinic visit rate

**Fig. 4:**
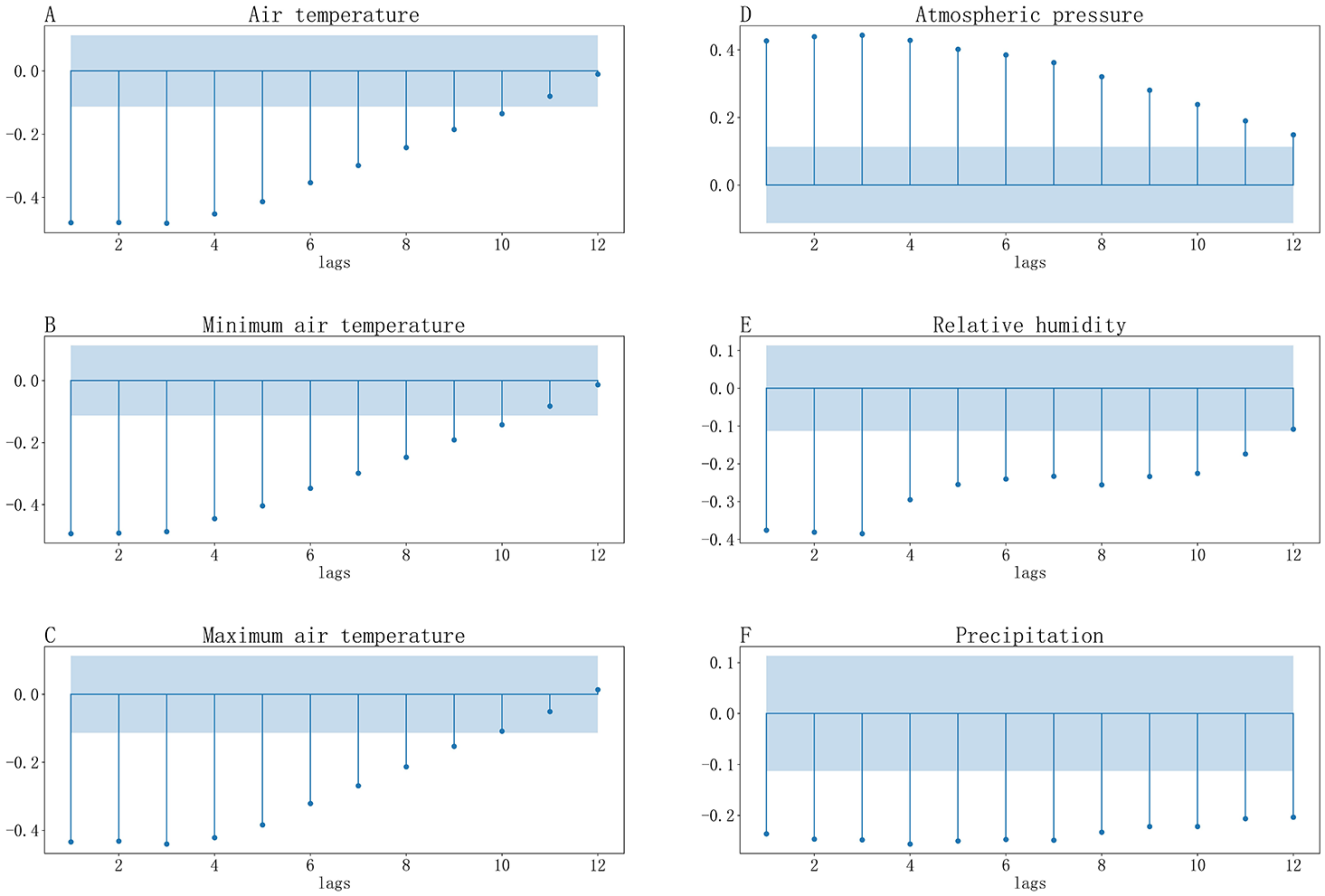
(A-F): Cross-correlation coefficients between weekly number of infectious diarrhea cases and (**A**) weekly mean air temperature, (**B**) weekly mean minimum air temperature, (**C**) weekly mean maximum air temperature, (**D**) weekly mean atmospheric pressure, (**E**) weekly mean relative humidity, (**F**) weekly mean precipitation

Figure 2 showed that the autocorrelation coefficients of the weekly number of infectious diarrhea cases from 1-week to 8-week lags were significant. Figure 3 indicated that the weekly number of infectious diarrhea cases was significantly correlated with weekly number of gastroenterology outpatient clinic visits from 1-week to 11-week lags, and was significantly correlated with weekly gastroenterology outpatient clinic visit rate from 1-week to 5-week lags. We could see from Fig. 4 that the weekly number of infectious diarrhea cases was also significantly associated with the meteorological factors including weekly mean air temperature, weekly mean minimum air temperature, weekly mean maximum air temperature, weekly mean atmospheric pressure, weekly mean relative humidity, and weekly mean precipitation from 1-week to 9-week lags.

### Feature selection and performance of base models

According to the results of the autocorrelation and cross-correlation analyses described above, the values of n in Eq. (1) were therefore set to 2, 3, 4, and 5, respectively. Since none of the models we used were susceptible to collinearity, both the inclusion as well as the non-inclusion of other predictors: weekly number of outpatient visits and weekly outpatient visit rate, each meteorological variable, were considered in the construction of the four base prediction models (see Table 1 for the symbols of each variable). The results for the comparison of model prediction performance incorporating historical data and other predictors are shown in Table 2.

Table 2 depicted the best RMSEs, MAEs, and MAPEs of the four base models, ANN, LSTM, SVR, and XGBoost, for various feature selections, with the variables they incorporated accordingly. The base models were initiated with the inclusion of the weekly number of infectious diarrhea cases with a time lag of some weeks, and then successively including the outpatient variables and meteorological variables. Overall, among ANN, LSTM, and SVR, models that incorporated the outpatient variables and weekly number of infectious diarrhea cases were able to achieve lower RMSEs, MAEs, and MAPEs than models that added meteorological variables, differing only in the time lags of the incorporated variables. Specifically, the time lags n of the variables included in ANN, LSTM, SVR and XGBoost were 2, 5, 5 and 5, respectively.

In terms of RMSE, MAE, and MAPE, the LSTM had the best prediction performance among the four base models. Specifically, the minimum RMSE, MAE, and MAPE of ANN were: 86.57, 60.47 and 16.83%; the minimum RMSE, MAE, and MAPE of LSTM were: 84.85, 57.50 and 15.92%; the minimum RMSE, MAE, and MAPE of SVR were: 86.41, 61.31 and 16.37%; the minimum RMSE, MAE, and MAPE of XGBoost were: 85.13, 63.12 and 17.25%.

**Table 1:** The variables and their corresponding symbols

Variable name	Symbol
weekly number of infectious diarrhea cases	*N*_*cases*_
weekly number of gastroenterology outpatient clinic visits	*N*_*clinic−visits*_
weekly gastroenterology outpatient clinic visit rate	*Rate*_*clinic*_
weekly mean air temperature	*T*
weekly mean minimum air temperature	*T*_*min*_
weekly mean maximum air temperature	*T*_*max*_
weekly mean atmospheric pressure	*Pressure*
weekly mean relative humidity	*RH*
weekly mean precipitation	*Precipitation*

**Table 2:** The minimum RMSEs, MAEs and MAPEs of testing set for ANN, LSTM, SVR, and XGBoost and their included variables

Model	Variables included in the model	RMSE	MAE	MAPE
ANN	***N***_***cases***_	95.52	66.56	18.20%
	***N***_***cases***_, *N*_*clinic-visits*_ and *Rate*_*clinic*_	86.57	60.47	16.83%
	***N***_***cases***_, *T*, *T*_*min*_, *T*_*max*_, *Pressure*, *RH*, and *Precipitation*	90.65	69.91	19.46%
	***N***_***cases***_, *T*, *T*_*min*_,*T*_*max*_, *Pressure*, *RH*, *Precipitation*, *N*_*clinic-visits*_ and *Rate*_*clinic*_	107.03	78.99	20.42%
LSTM	***N***_***cases***_	88.67	57.50	16.38%
	***N***_***cases***_, *N*_*clinic-visits*_ and *Rate*_*clinic*_	84.85	59.07	15.92%
	***N***_***cases***_, *T*, *T*_*min*_, *T*_*max*_, *Pressure*, *RH*, and *Precipitation*	92.71	68.21	20.13%
	***N***_***cases***_, *T*, *T*_*min*_, *T*_*max*_, *Pressure*, *RH*, *Precipitation*, *N*_*clinic-visits*_ and *Rate*_*clinic*_	95.35	66.25	18.37%
SVR	***N***_***cases***_	91.78	61.74	16.55%
	***N***_***cases***_, *N*_*clinic-visits*_ and *Rate*_*clinic*_	86.41	61.31	16.37%
	***N***_***cases***_, *T*, *T*_*min*_, *T*_*max*_, *Pressure*, *RH*, and *Precipitation*	121.04	92.26	29.22%
	***N***_***cases***_, *T*, *T*_*min*_, *T*_*max*_, *Pressure*, *RH*, *Precipitation*, *N*_*clinic-visits*_ and *Rate*_*clinic*_	121.38	93.70	29.42%
XGBoost	***N***_***cases***_	93.43	63.12	17.31%
	***N***_***cases***_, *N*_*clinic-visits*_ and *Rate*_*clinic*_	89.19	66.98	18.70%
	***N***_***cases***_, *T*, *T*_*min*_, *T*_*max*_, *Pressure*, *RH*, and *Precipitation*	88.00	63.61	17.25%
	***N***_***cases***_, *T*, *T*_*min*_, *T*_*max*_, *Pressure*, *RH*, *Precipitation*, *N*_*clinic-visits*_ and *Rate*_*clinic*_	85.13	63.77	18.76%

### Prediction performance of stacking

**Fig. 5:**
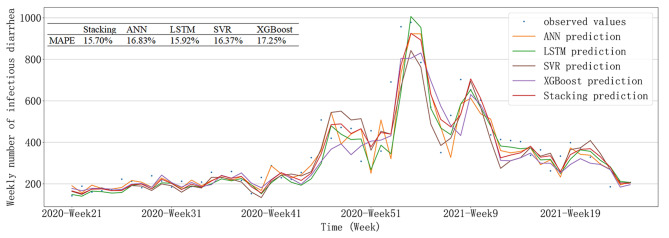
Time-series plots of predicted values compared to observed values based on testing set

Since the model structure and feature selection differed among the four base models when achieving the minimum RMSE, MAE, and MAPE, we used three approaches in the selection of base models to implement stacking: selecting the base models with the lowest RMSE, selecting the base models with the lowest MAE and selecting the base models with the lowest MAPE. Table 3 presents the prediction performance of the stacking ensembled models and their corresponding base models. As shown in Table 3, the predictive performance of stacking models was often better than those of base models. In some cases, the advantage in predictive performance of stacking models was not significant, but the predictive performance of the stacking model was always close to the best-performing model of the base models. The optimal stacking ensembled model was the one ensembled by the base models of the lowest MAPE, whose RMSE, MAE, and MAPE were 75.82, 55.93, and 15.70%, respectively. Figure 5 mainly showed the comparison between the prediction values and the observed values. It could be seen that the predictions of these models differed little from the observed values and were effective in predicting wave peaks.

**Table 3:** Prediction performance of stacking ensembled model

Selection of base models	Model names	RMSE	MAE	MAPE
The base models with the lowest RMSE	ANN	86.57	60.47	17.38%
	LSTM	84.85	59.07	16.99%
	SVR	86.41	61.31	16.37%
	XGBoost	85.13	65.75	19.16%
	Stacking	74.38	55.22	16.01%
The base models with the lowest MAE	ANN	86.57	60.47	17.38%
	LSTM	88.67	57.50	16.52%
	SVR	86.41	61.31	16.37%
	XGBoost	93.43	63.12	17.31%
	Stacking	77.70	58.54	16.88%
The base models with the lowest MAPE	ANN	92.75	63.13	16.83%
	LSTM	90.90	59.07	15.92%
	SVR	86.41	61.31	16.37%
	XGBoost	88.00	63.61	17.25%
	Stacking	75.82	55.93	15.70%

*Note* The underlined model was the best stacking ensembled model according to MAPE

## Discussion

This study used stacking ensemble learning to build a prediction model for infectious diarrhea in Guangzhou. The four base models (ANN, LSTM, SVR, and XGBoost) were ensembled together, and the prediction performance of the final model was improved compared to each base model. The results indicated that stacking ensemble learning could alleviate the difficulty in optimal model selection. We also explored the application of symptom surveillance data in the prediction of infectious diarrhea, which could effectively enhance the performance of predictive models. Our research could serve as a valuable reference for developing predictive models and utilizing multi-source data.

There was a significant correlation between the outpatient variables and the weekly number of infectious diarrhea cases. The meteorological factors of air temperature, atmospheric pressure, relative humidity, and precipitation were also significantly correlated with the number of infectious diarrhea cases. Theoretically, patients attending the gastroenterology outpatient clinic included some patients who might not have infectious diarrhea, and not all patients diagnosed with infectious diarrhea were identified through the gastroenterology outpatient clinic. Therefore, the two should have some correlation, although they would not be completely correlated. As for the meteorological factors, it might be attributed to the fact that meteorological factors have an impact on some pathogenic organisms of infectious diarrhea and also on the susceptibility of the human body [28–30]. In general, both the outpatient variables and meteorological variables were reasonable predictors in the model construction.

We found that the outpatient variables from symptom surveillance data were more effective than the meteorological variables in improving the model prediction performance. In ANN, LSTM, and SVR models, the addition of outpatient variables to the initial model which only incorporated the lagged weekly number of infectious diarrhea cases could lead to a substantial improvement in model prediction performance. The temporal trends of meteorological variables suggested that the air temperature, atmospheric pressure, relative humidity, and precipitation in Guangzhou were almost consistent each year, with little variability (see Additional file 1: Figure S2 in Supplementary material). Whereas, outpatient variables showed more similar trends to the number of infectious diarrhea cases. In previous studies, it has also been demonstrated that the contribution of meteorological variables to the prediction of infectious diseases varied due to the different climates in different regions [31–33]. Moreover, previous studies have not incorporated predictors other than meteorological factors [3, 4], so whether other predictors (outpatient surveillance data, for example) are more effective than meteorological factors in prediction models have not been fully evaluated. Therefore, it was acceptable that the model performed better when outpatient variables alone were included. We found that using symptom surveillance data as predictors for infectious diarrhea was more effective than using meteorological factors in improving the predictive performance of the models. It can be a reference for subsequent researchers on how to construct multi-source databases that can be used for infectious disease prediction, and also can encourage further improvements in infectious disease surveillance to provide more usable data in the future. For instance, some smartphone apps could help acquire data directly from individuals rather than healthcare institutions and could expand basic health monitoring of the general population [34, 35].

Among the four base models ANN, LSTM, SVR, and XGBoost, LSTM had the best prediction performance, and the ensemble of the four base models by stacking could make the prediction performance further improved. However, from the perspective of RMSE, MAE, and MAPE, the prediction performance of the four base models did not differ much, and the prediction performance was influenced by the selected features. The reason for the better performance of LSTM is probably that it is a neural network especially for processing serial data, which can capture the features of time series more effectively [36]. LSTM model also performed better in many applications of infectious diseases prediction [18, 37, 38]. Although LSTM may be a relatively better prediction model, some studies showed different conclusions in the comparison of the prediction performance of different models [23, 39]. It might be due to differences in data and prediction goals. Accordingly, it is relatively difficult to select a model that is optimal in every scenario. Therefore, we have adopted the stacking ensemble method to integrate the base models. In this study, it could have at least two advantages: first, it could solve our difficulty in choosing a better model to some extent by combining some base models together; second, it could further promote the predictive performance of the model compared to the base models, and obtain a better model. The stacking ensemble method is currently used in many domains and competitions such as hydrological forecasts [40, 41], wind power forecasts [42], crash prediction [19] and influenza incidence prediction [21, 43], and it has shown some excellent performance. It can be seen from this study that the method of stacking ensemble can be applied more to the field of infectious disease prediction in future.

From the perspective of public health, one of the purposes of constructing a short-term prediction model is to provide advanced warning information to inform the decision-making of public health policies [44, 45]. In this study, we used the predictors with time lags, so the predicted values would be prior to the official statistics on confirmed cases. In addition, the predicted value would be compared with the warning threshold. A warning message would be issued when the predicted value was higher than the warning threshold. The early warning threshold values are often calculated by the moving average percentile control chart method [46], so the threshold values are based on historical number of cases, rather than values that are not yet observed. Therefore, in real-world situations, our predictive models can provide helpful information for public health decision-making.

There were some limitations in this study. The final model constructed in this study was actually a black box, so it could not analyze the importance of predictors or further explore the causal relationship between predictors and outcomes. We used testing data rather than data from a different region for external validation, so the results should be interpreted with caution when used in other areas. For research purposes, however, it already achieved our tasks for prediction. In addition, there might be more data that could be incorporated into the model as predictors, such as drug sales in pharmacies, the number of absentees in schools, etc. However, due to the difficulty in obtaining these data, this study did not include predictors other than outpatient variables and meteorological variables.

## Conclusions

The incorporation of symptom surveillance data had the potential to improve the predictive accuracy of infectious diarrhea prediction models, and symptom surveillance data was more effective than meteorological data in enhancing model performance. Furthermore, by using the stacking method to combine multiple prediction models, we were able to alleviate the difficulty in selecting the optimal model, and could obtain a model with better performance than base models. Further improvements in infectious disease surveillance will generate more usable data, which can facilitate the development of more accurate prediction models. In addition, the stacking method could also be implemented more in the construction of future infectious disease prediction systems.

### Electronic supplementary material

Below is the link to the electronic supplementary material.

Supplementary Material 1

## Data Availability

The datasets analyzed during the current study are not publicly available due to privacy and regulation, but may be available from Guangzhou center for disease control and prevention and from corresponding author upon reasonable request.

## Abbreviations

GBD: Global Burden of Disease
DALYs: Disability-adjusted life years
ARIMA: The autoregressive integrated moving average model
ANN: Artificial neural networks
LSTM: Long Short-Term Memory networks
SVR: Support vector regression
XGBoost: Extreme gradient boosting regression trees
MAPE: Mean absolute percentage error
RMSE: Root mean square error
MAE: Mean absolute error
