# Supplementary material for: Predicting the incidence of infectious diarrhea with symptom surveillance data using a stacking-based ensembled model
Source: BMC Infect Dis. 2024 Feb 26;24:265. doi: 10.1186/s12879-024-09138-x (PMC10898154; doi:10.1186/s12879-024-09138-x)
Supplement: Supplementary file 1 — Supplementary Material 1 [file 12879_2024_9138_MOESM1_ESM.docx]

### Supplementary material


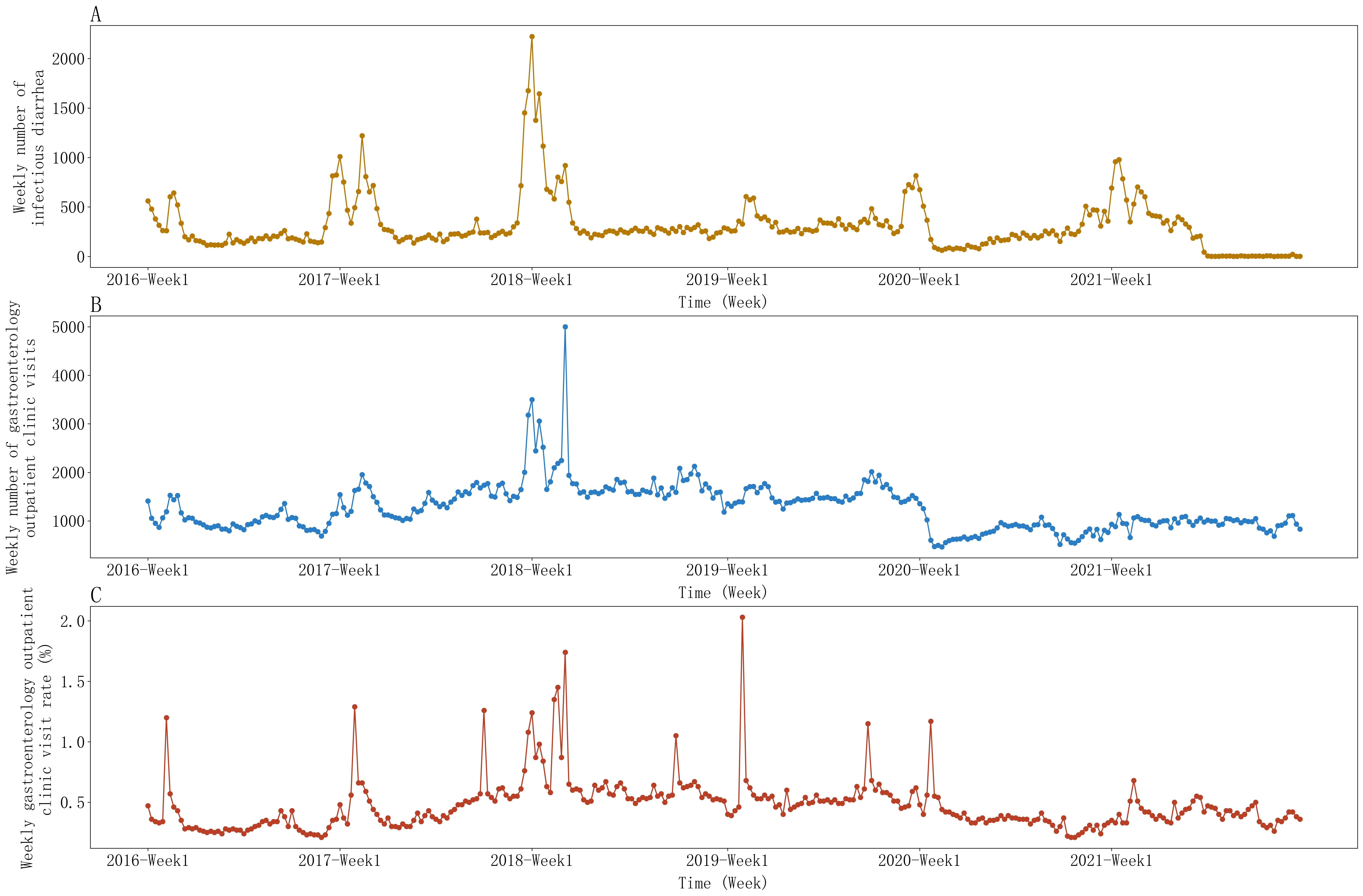


**Figure S1** (A)-(C): (A) Weekly number of infectious diarrhea cases (B) Weekly number of gastroenterology outpatient clinic visits (C)Weekly gastroenterology outpatient clinic visit rate in Guangzhou from 2016 to 2021.

Note: we found that in the descriptive analysis, the mean number of infectious diarrhea cases from June 2021 to Dec 2021 was only 6 per week, whereas the average number of infectious diarrhea cases per week from Jan 2016 to Dec 2021 was 311. It could be seen from Figure S1 that the series after June 2021 also showed an unusual sharp decline. From the statistical characteristics of the data, the data of infectious diarrhea cases after June 2021 (weeks 26 to 52 of 2021) could be regarded as outliers. Our communication with relevant collaborators and searching for relevant information revealed that it was due to a COVID-19 outbreak in Guangzhou in June 2021 that affected related work, resulting in an inaccurate number of infectious diarrhea cases. As a result, we excluded data after June 2021 to eliminate the possible effect of outliers.





**Figure S2**. Meteorological variables: (A) weekly mean air temperature, (B) weekly mean minimum air temperature, (C) weekly mean maximum air temperature, (D) weekly mean atmospheric pressure, (E) weekly mean relative humidity, and (F) weekly mean precipitation in Guangzhou from 2016 to 2021.
